# Supplementary figures and images for: Unveiling functions of the visual cortex using task-specific deep neural networks
Source: PLoS Comput Biol. 2021 Aug 13;17(8):e1009267. doi: 10.1371/journal.pcbi.1009267 (PMC8407579; doi:10.1371/journal.pcbi.1009267)

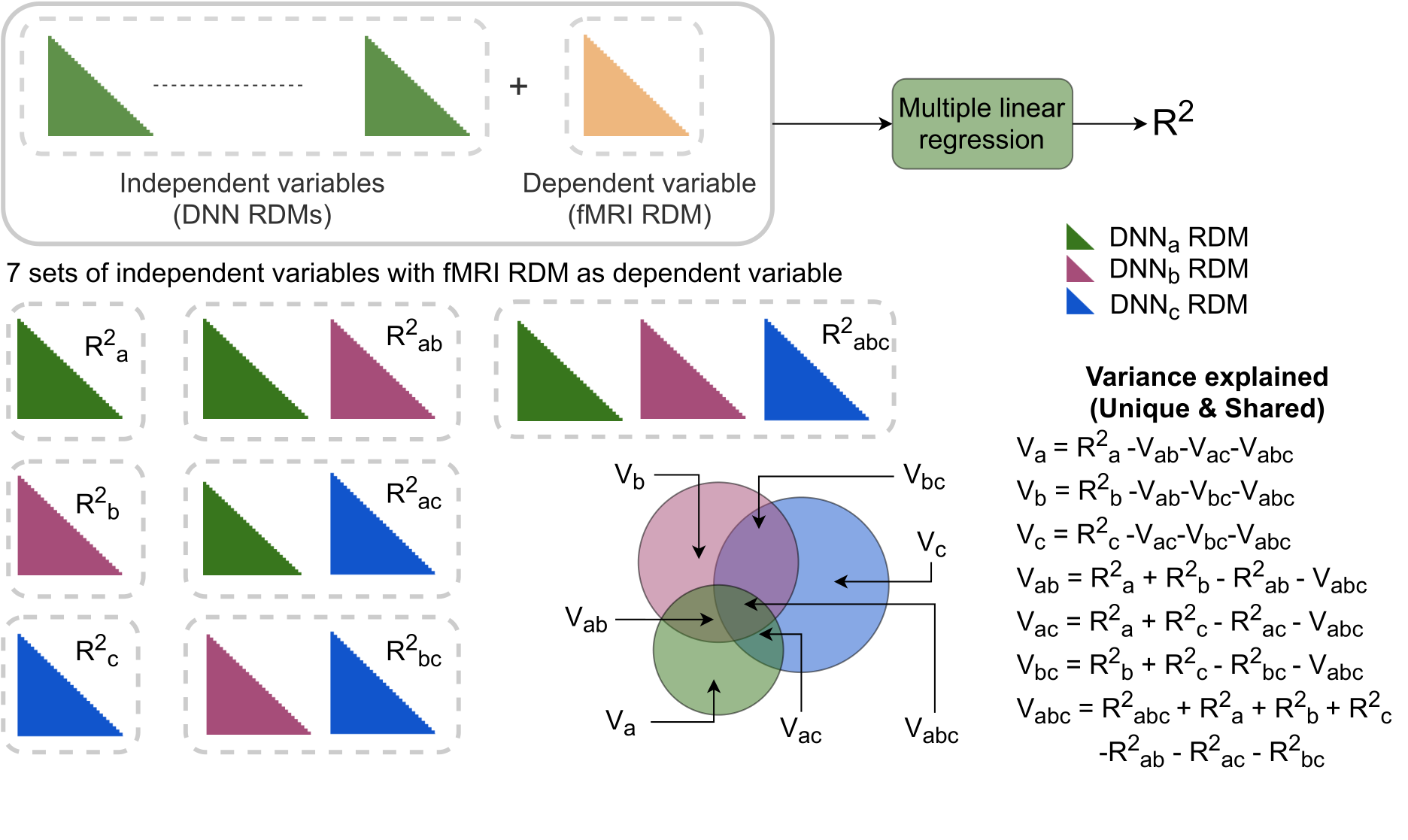

Supplement: S1 Fig — Given a set of multiple independent variables and dependent variables, multiple linear regression results in R-squared (R2) that represents the proportion of the variance for a dependent variable that’s explained by independent variables in a regression model. To find how 3 DNN RDMs together explain the variance of a given fMRI RDM we perform 7 multiple regression and illustrate unique and shared variance explained by models through a Venn diagram. (TIFF) [file pcbi.1009267.s001.tiff]

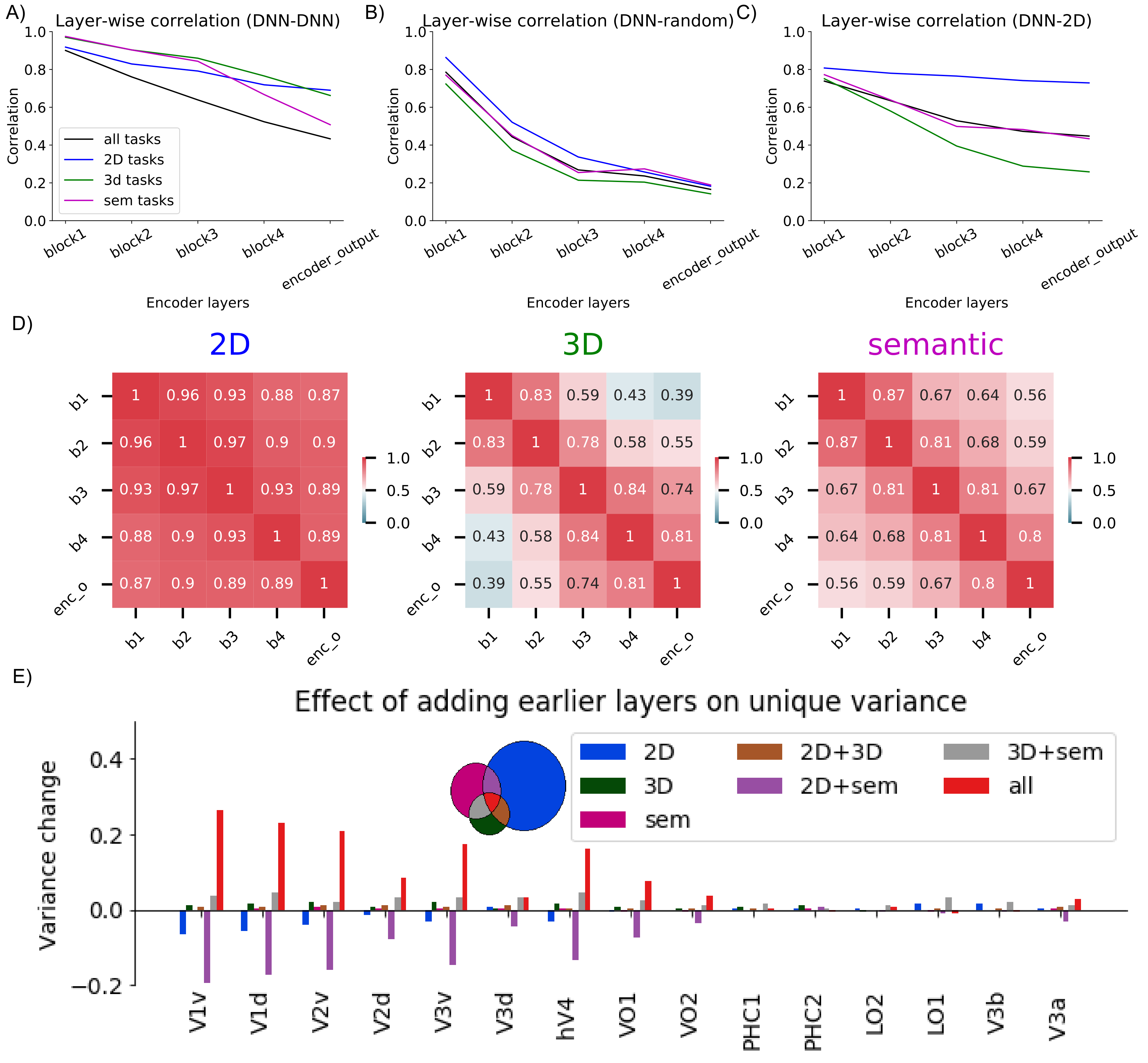

Supplement: S2 Fig — A) Spearman’s correlation of all DNN RDMs at a given layer of the encoder with other DNN RDMs computed at the same layer. We report the mean pairwise correlation of all 18 DNNs at different layers of the encoder. B) Spearman’s correlation of all DNN RDMs at a given layer of the encoder with a randomly initialized model with the same architecture computed at the same layer. We report the mean correlation of all 18 DNNs with the randomly initialized DNN at different layers of the encoder. C) Spearman’s correlation of all DNN RDMs at a given layer of the encoder with deeper layers (block4 and encoder output) of 2D DNNs. We report the mean correlation of the key layers of all 18 DNNs with deeper layers (block4 and encoder output) of 2D DNNs. D) Spearman’s correlation between layers at different depths for DNNs corresponding to different task types. We report the mean correlation between different layers averaged across different DNNs of the same task type. E) Effect of adding all the key layers on unique and shared variance of fMRI RDMs from different ROIs as compared to selecting only task-specific layers for variance partitioning analysis. We report the change in variance explained (variance change) for 7 variance partitions when all key layers were used for analysis as compared to selecting task-specific layers. (TIFF) [file pcbi.1009267.s002.tiff]

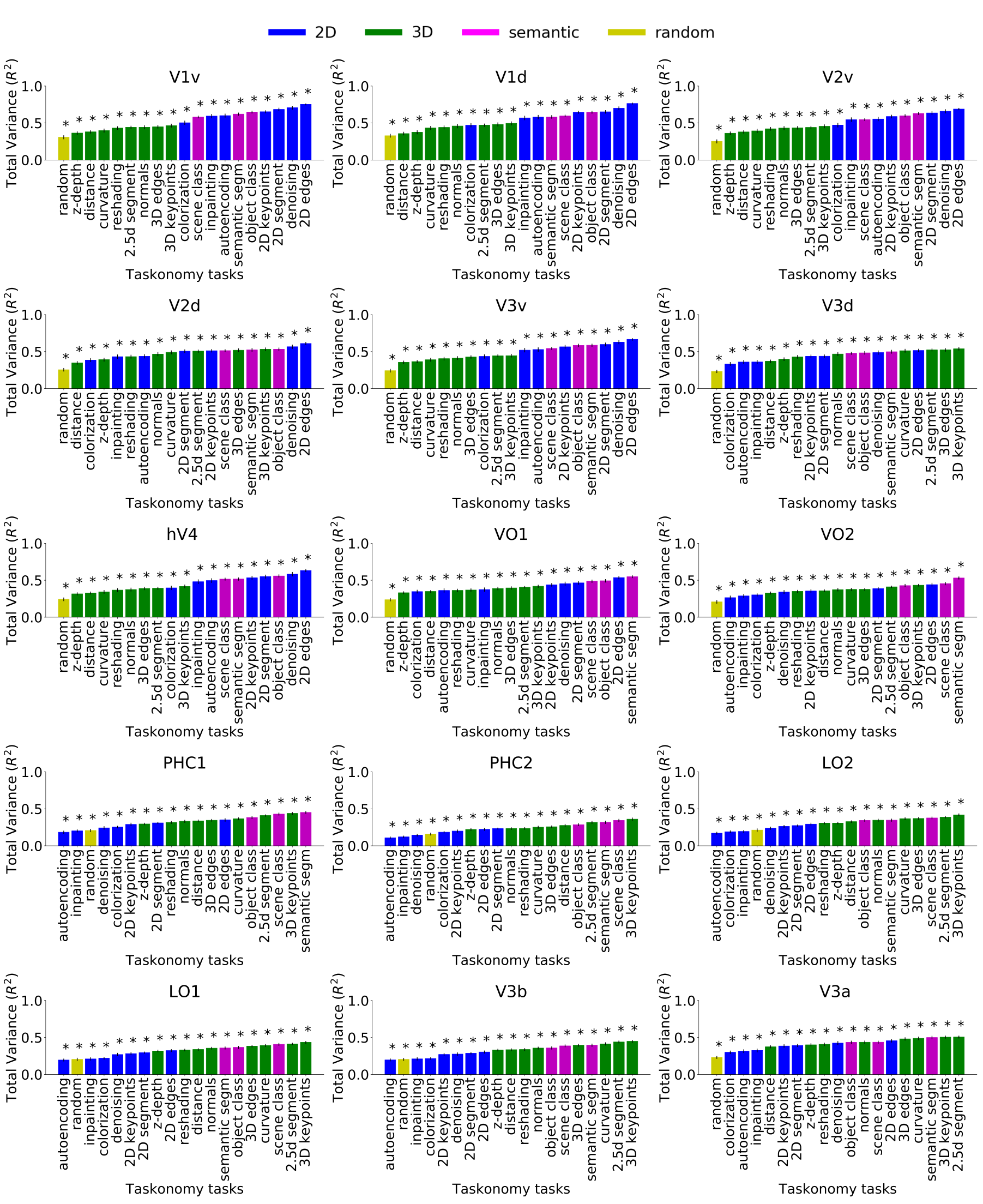

Supplement: S3 Fig — The bar plot shows the absolute total variance of each ROI RDM explained by task-specific layer RDMs of a given DNN. The asterisk denotes the significance of total variance (p<0.05, permutation test with 10,000 iterations, FDR-corrected across DNNs). The error bars show the standard deviation calculated by bootstrapping 90% of the conditions (10,000 iterations). (TIFF) [file pcbi.1009267.s003.tiff]

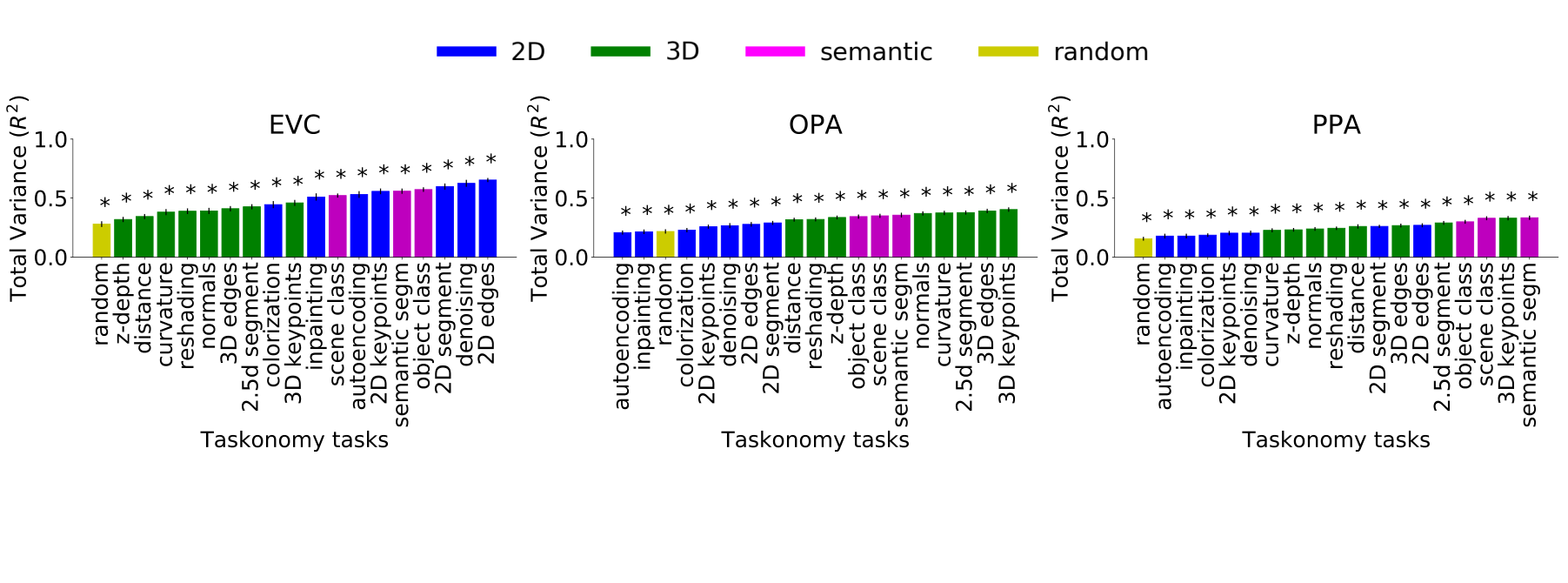

Supplement: S4 Fig — The bar plot shows the absolute total variance of each ROI RDM explained by task-specific layer RDMs of a given DNN. The asterisk denotes the significance of total variance (p<0.05, permutation test with 10,000 iterations, FDR-corrected across DNNs). The error bars show the standard deviation calculated by bootstrapping 90% of the conditions (10,000 iterations). (TIFF) [file pcbi.1009267.s004.tiff]

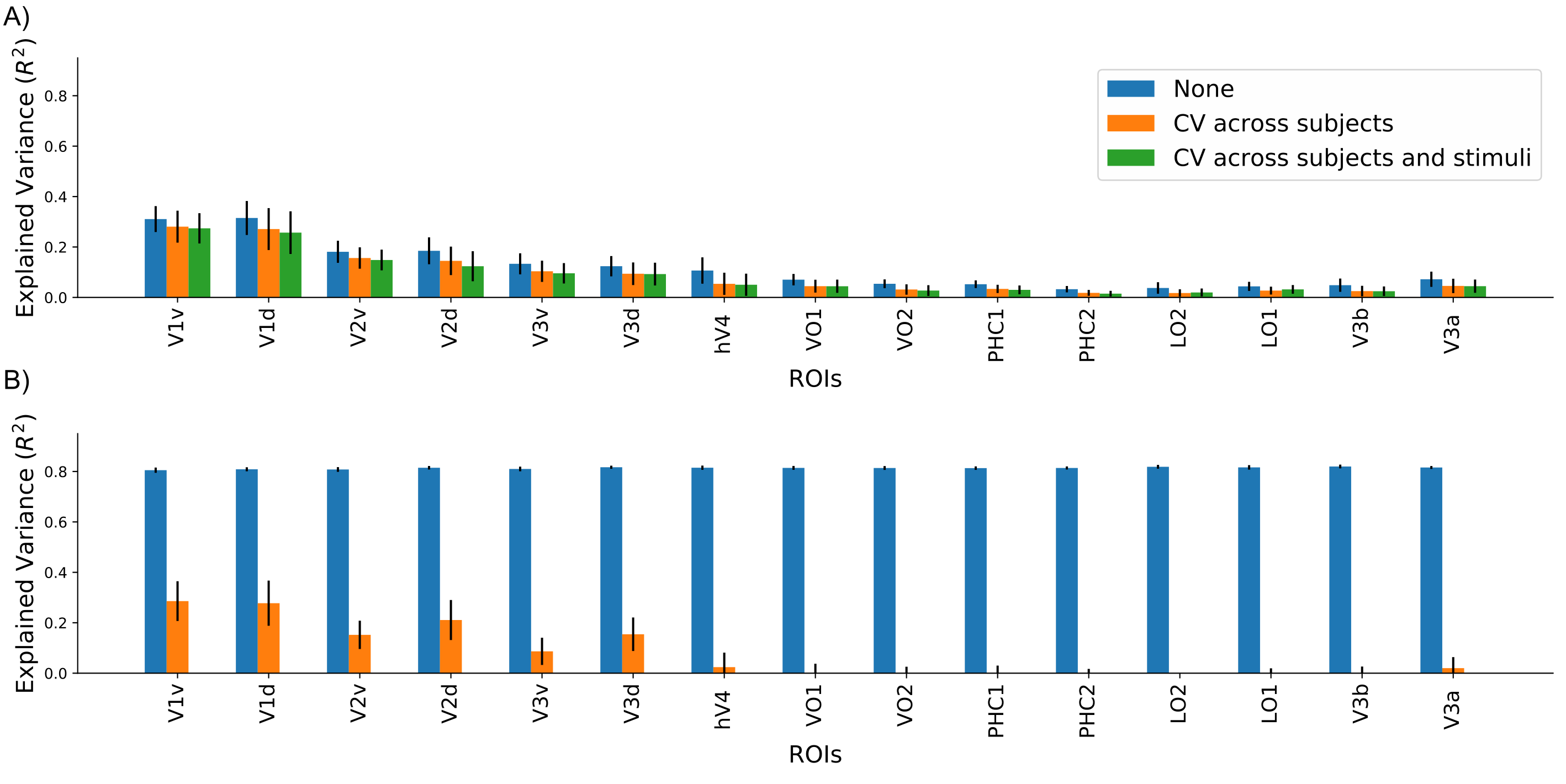

Supplement: S5 Fig — A) Variance of each ROI explained by top-3 best predicting DNNs compared for different cross-validation settings (blue bars: no cross validation; orange bars: cross validation across subjects; green bars: cross validation across subjects and stimuli). The error bars show the 95% confidence interval calculated across N = 16 subjects. All the R2 values are statistically significant (p<0.05, two-sided t-test, FDR-corrected across ROIs) B) Variance of each ROI explained by 1000 randomly generated RDMs compared for different cross-validation settings (blue bars: no cross validation; orange bars: cross validation across subjects; green bars: cross validation across subjects and stimuli). The error bars show the 95% confidence interval calculated across N = 16 subjects. (TIFF) [file pcbi.1009267.s005.tiff]
