# Supplementary material for: Unveiling functions of the visual cortex using task-specific deep neural networks
Source: PLoS Comput Biol. 2021 Aug 13;17(8):e1009267. doi: 10.1371/journal.pcbi.1009267 (PMC8407579; doi:10.1371/journal.pcbi.1009267)
Supplement: S1 Text — (DOCX) [file pcbi.1009267.s006.docx]

## Selecting Task-specific DNN representations

Our aim was to select the layers of the encoders of the DNN that had task-specific representation. By task-specific representation, we refer to representation learned by the DNN to perform the corresponding task. We performed multiple analyses to find out which layers of the encoder consisted of the most task-specific information. In the first analysis, we calculated the Spearman’s correlation of one DNN RDM from a given layer with all the other DNN RDMs from the same layer.

We performed this analysis for all pairwise combinations of DNNs investigated in this study and plotted the mean correlation for all pairwise DNN comparisons per layer in S3A Fig. In S3A Fig, we observed that early layers of the encoder showed a higher mean pairwise correlation than the deeper layers. The results suggest that early layers of DNN learn similar representation irrespective of the task DNN was optimized for, while task-specificity increases as we go deeper in the network. In the second analysis, we calculated the Spearman’s correlation of RDMs of a given layer from all the 18 DNNs investigated in this study and compared with the RDM of the same layer from a randomly initialized network having the exact same encoder architecture (S3B Fig). In S3B Fig, we observed that early layers showed a higher correlation with randomly initialized DNN than deeper layers. The results reinforce our argument that early layers learn a general representation irrespective of the task DNN was optimized for while deeper layers consist of more task-specific information.

An arguably attractive procedure for layer selection is to select all key layers for each of the DNNs and then perform the comparison. We argue against this by performing an analysis comparing the representation of late layers of 2D DNNs (block 4 and encoder output) with key layers of all the DNNs (S3C Fig). We find that early layers of all the DNNs show a high correlation with late layers of 2D DNNs, suggesting that early layers of all DNNs learn a representation required to perform low-level 2D tasks irrespective of the tasks they need to perform (3D or semantic). We further validate this argument by comparing the correlation between different layers of DNNs within a task type (S3D Fig). We find that in 2D DNNs the late layers show a high correlation with early layers, suggesting that to perform 2D functions DNNs learn very similar representations at different depths of the network. In the case of 3D and semantic DNNs, the late layers show low correlation with early layers, suggesting that a different representation is required to perform these tasks and that these representations are found in late layers.

The early layer representations of all DNNs are very similar to representations learned by 2D DNNs. Including these layers into the variance partitioning analysis could diminish the unique variance of fMRI RDMs explained by 2D DNNs due to an increase in shared variance explained by all the DNNs together. We show the above effect by reporting the change in unique and shared variance when all key layers were used in variance partitioning analysis corresponding to Section 3 of main text instead of the last 2 layers of the encoder (S3E Fig). We observe that adding early layers of all 3 different types of DNNs in the analysis leads to an increase in shared variance explained by all these models together and reducing the unique variance contribution of 2D DNNs significantly in the early visual regions. We further observe that in high-level ROIs for which the unique variance of 2D DNNs was insignificant in the original analysis, we barely notice any changes in the unique variance explained. Therefore, to observe the differences in the DNNs due to the task they were optimized to perform we selected the last two layers of the DNNs as the task-specific representation.
