# Supplementary material for: Unveiling functions of the visual cortex using task-specific deep neural networks
Source: PLoS Comput Biol. 2021 Aug 13;17(8):e1009267. doi: 10.1371/journal.pcbi.1009267 (PMC8407579; doi:10.1371/journal.pcbi.1009267)
Supplement: Attachment — Submitted filename: Response_to_reviewers.docx [file pcbi.1009267.s007.docx]

# Summary of the Response

We thank all the reviewers for their constructive feedback and insightful suggestions. Please find the responses (in black font) to each of the reviewer’s comments (*in blue italic font*). We have also added here in *italics* the text excerpts from the revised manuscript that incorporate the suggested changes.

***Reviewer #1:***

*In their manuscript, the authors compare task-specific CNNs to regions along the visual cortex to investigate their respective functionalities. The idea is that if a cortical region is involved in a specific task, it will show a stronger mapping to a CNN trained on a similar task compared to a CNN trained on a different task. The authors find that CNNs trained on low-level visual tasks better mapped to early brain regions, 3D-perceptual tasks to dorsal regions and semantic tasks regions in the ventral stream. I would further like to point out that the authors did a great job in setting up a website demonstrating their results with visualizations, etc. I really appreciated it! Overall, while the approach seems novel and promising and the data support coarse differences in functionality of different cortical regions, I have several concerns regarding the limitation of the tasks and neural data and the novelty of the findings per se.*

We thank the reviewer for appreciating the interactive visualizations and considering our work a promising novel approach. We address the reviewer's specific concerns in detail below.

*Main comments:*

*1) My first comment concerns the generalizability and interpretation given the tasks and fMRI data are both limited to scene perception. Overall, I think the paper would be stronger if something could be added in terms of the functionality within cortical areas that are related to scene perception than trying to map these tasks/stimuli to the entire cortex. To give more details:*

*a. The authors focus on a set of CNNs trained on scene-related tasks based on the Taskonomy dataset. It is a great to have a set of different tasks for the same dataset, but these tasks have not been chosen to be biologically relevant and many of them seem less ecological relevant (e.g., normal, autoencoding, etc). The choice of tasks will affect the conclusion of what cortical area might be doing which task. The authors summarized those tasks as 2D, 3D and semantic. I agree that these coarse distinctions seem relevant for visual processing but it is hard to know what would happen if a different set of tasks was used for the analysis (e.g., subordinate, superordinate categorization, next-frame prediction, etc.), especially for areas which are not involved in scene processing. The authors discuss this limitation briefly but then don’t discuss the consequences for the interpretation of functionality in regions that are most likely not involved in scene perception. This needs to be added.*

The reviewer's key concern is how to interpret the functionality in non-scene areas based on the current choice of tasks. We agree with the reviewer’s point that the choice of task will affect the interpretation of the functionality of the brain region. In the current version of the manuscript we therefore state in the discussion that the interpretation of regional functions in our study are limited by the choice of tasks investigated. In our view the functions found for the non-scene areas will correspond to what these regions might be doing when humans perceive scenes. In contrast, we make no claims about the functions of those brain regions during the perception of objects, faces, bodies or other types of images. As suggested by the reviewer we have clarified this in the discussion section. Please find the excerpt from the revised manuscript highlighting the above point.

**Excerpts from revised manuscript Page 17, Lines 508 to 512**

*“A limitation of our study is that our findings are restricted to functions related to scene perception. Thus, the functions we discovered for non-scene regions correspond to their functions when humans are perceiving scenes. In contrast, our study does not characterize the functions of these regions when humans perceive non-scene categories such as objects, faces, or bodies.”*

*b. The fMRI dataset used in the manuscript was useful as it well fitted the CNNs’ training diet and was related to scene perception. However, we know that scene perception activates only a certain part of the human visual cortex (mainly RSC, OPA, PPA). Thus, this dataset and the fMRI experiment were optimized to achieve strong responses in early visual and scene-processing areas but most likely did not result in strong activation in many other areas (e.g., EBA, FFA, STS to name a few). This is reflected in the low noise ceiling achieved in these areas suggesting that there is not much signal to begin with. I think it is difficult to interpret the mapping between CNNs and areas that are barely responding to the stimuli. In my opinion, given this dataset, it would make more sense to focus on (ideally functionally-localized) areas related to scene processing and to test which task best explains voxels in PPA or OPA for example. There are still many open questions to a subdivision in PPA, the differences between OPA and PPA and others that could be investigated. If the authors could stand it, I think the paper would greatly benefit from looking at differences in those areas. In particular since the PPA, OPA, and RSC have all been functionally defined in the fMRI experiment the manuscript is based on (Bonner & Epstein, 2017). Without such an analysis, I am not sure what novel contribution the manuscript does on the functionality of the visual cortex, beyond introducing a novel method (It is of course fine to introduce a novel method but that would need restructuring and rephrasing the manuscript).*

We thank the reviewer for suggesting the above additional analysis. We have added a new, full section in the manuscript about functionally localized scene-selective regions (OPA and PPA; RSC was excluded due to low noise ceiling; EVC was included as a control) that were investigated in Bonner and Epstein 2017. We show the results using top-3 best predicting DNNs and grouped DNNs in Rebuttal Figure 1 (Figure 4 in the manuscript) below. We find different functional roles of these ROIs. We discuss these findings in detail in the excerpt from the revised manuscript below.

**Rebuttal Figure 1 (Figure 4 in revised manuscript):** **Functional roles of localized ROIs**: **a)** Absolute total variance (R^2^) explained in functionally localized ROIs by using the top-3 DNNs together. The Venn diagram for each ROI illustrates the unique and shared variance of the ROI responses explained by the combination of the top-3 DNNs. The bar plot shows the unique variance of each ROI explained by each of the top-3 DNNs individually. The asterisk denotes the significance of unique variance and the difference in unique variance (p<0.05, permutation test with 10,000 iterations, FDR-corrected across DNNs). The error bars show the standard deviation calculated by bootstrapping 90% of the conditions (10,000 iterations). **b)** Absolute total variance (R^2^) explained in functionally localized ROIs by using 3 DNN RDMs averaged across task groups (2D, 3D, or semantic). The Venn diagram for each ROI illustrates the unique and shared variance of the ROI responses explained by the combination of 3 DNN task groups. The bar plot shows the unique variance of each ROI explained by each task group individually. The asterisk denotes whether the unique variance or the difference in unique variance was significant (p<0.05, permutation test with 10,000 iterations, FDR-corrected across DNNs). The error bars show the standard deviation calculated by bootstrapping 90% of the conditions (10,000 iterations).

**Excerpts from revised manuscript Page 12-13, Lines 353 to 384**

“*2.4 Functional roles of scene-selective regions*

*In the previous sections, we focused on discovering functions of regions anatomically defined by an atlas. Since the stimulus set used to record fMRI responses consisted of indoor scenes, in this section we investigate functional differences in functionally localized scene-selective regions. We here focus on two major scene-selective ROIs: occipital place area (OPA) and parahippocampal place area (PPA), putting results into context with early visual cortex (EVC) as an informative contrast region involved in basic visual processing. The analysis followed the general rationale as used before.*

*We first investigated the functional differences in these regions by performing variance partitioning analysis using top-3 DNNs (see R^2^ based ranking of all DNNs in supplementary Figure 3) that best explained a given ROIs’ responses (Figure 4a). We found that the DNN that detects edges explained significantly higher variance (p<0.05, permutation test, FDR-corrected) in EVC uniquely than the other two DNNs, suggesting a function related to edge detection. 3D DNNs (3D Keypoints, 2.5D Segmentation, 3D edges) were best predicting DNNs for OPA suggesting its role in 3D scene understanding. A combination of semantic (semantic segmentation, scene classification) and 3D (3D keypoints) DNNs were best predicting DNNs for PPA suggesting its role in both semantic and 3D scene understanding.*

*We then investigated the functional differences by performing variance partitioning analysis using aggregated 2D, 3D, and semantic DNN RDMs obtained by averaging the individual DNN RDMs in each task group (Figure 4b). We found that for EVC and OPA results are highly consistent with top-3 DNN analysis showing a prominent unique variance explained by the 2D DNN RDM in EVC and the 3D DNN RDM in OPA. Interestingly, in PPA we find that the semantic DNN RDM shows the highest unique variance with no significant unique variance explained by the 3D DNN RDM. The insignificant unique variance explained by the 3D DNN RDM is potentially due to averaging the DNN RDMs of all 3D DNNs (high ranked as well as low ranked) which may lead to diminishing the contribution of an individual high ranked 3D DNN RDM (e.g. 3D keypoints that was in top-3 DNNs for PPA). Overall, we find converging evidence that OPA is mainly related to tasks involved in 3-dimensional perception (3D), and PPA is mainly related to semantic (categorical) understanding of the scene.”*

**Excerpts from revised manuscript Page 15, Lines 464 to 467**

*“In scene-selective ROIs, we found a prominent semantic functional role for PPA and 3D functional role for OPA. Our study extends the findings of a previous study^51^ relating OPA and PPA to 3D models by differentiating between OPA and PPA functions through a much broader set of models.”*

*2) Related to the previous point, the noise ceiling in many of the cortical areas investigated is particularly low. Given a more diverse set of stimuli an R^2 of at least 0.1 in human IT can be achieved (e.g., Storrs et al., bioRxiv, 2020). The authors focus on the amount of explainable variance in the abstract and paper but given that the noise ceiling is so low this number is misleading (e.g., 60% of a highly noisy area is still low). This should be explicitly discussed.*

Please note that the numbers reported in the plot in Figure 5 of Storrs et al. biorXiv 2020 are R, not R^2^. For our study, if we plot R then the R values are above 0.1 in all these ROIs as can be observed in Rebuttal Figure 2 (not included in manuscript) below. To alleviate the reviewer's concern about misleading the readers by reporting only percent explainable variance, we mentioned minimum, median and maximum of the absolute cross-validated R^2^ values in the revised manuscript. Please find the excerpt from the revised manuscript below.

**Rebuttal Figure 2 (not included in manuscript):** **Comparison to noise ceiling (R)**: Pearson’s correlation (R) between fitted RDM using linear combination of top-3 best predicting DNN RDMs (cross validated across subjects and conditions) and ROI RDM indicated in blue bars compared with lower and upper bound of noise ceiling indicated by shaded gray region. The error bars show the 95% confidence interval calculated across N=16 subjects. All the R values are statistically significant (p<0.05, two-sided t-test, FDR-corrected across ROIs).

We would also like to add that the noise ceiling is comparable to related study investigating scene selective regions (Groen et al. 2018)

**Excerpts from revised manuscript Page 9, Lines 260 to 266**

*“We found that variance explained in nine ROIs (V1v, V1d, V2v, V3v, VO1, PHC1, LO2, LO1, V3a) is higher than 60% of the lower bound of noise ceiling (Figure 2c, absolute R^2^ = 0.085 ± 0.046). In absolute terms, the minimum, median and maximum cross-validated R^2^ values across the 15 ROIs were 0.014 (PHC2), 0.044 (VO1) and 0.27 (V1v) which are comparable to related studies^60^ performing evaluation in a similar manner. This shows that the DNNs selected in this study predict fMRI responses well and therefore are suitable for mapping the functions of the investigated ROIs.”*

*3) The authors map the last two layers of the encoder of the CNNs to the fMRI data. They motivate this decision by the finding that representations in earlier stages of processing in the CNNs are not task-specific but shared across CNNs. However, as the authors describe in the manuscript, the visual cortex is build up in a hierarchy where later stages of processing build on earlier stages of processing. So it seems odd that to map an encoder output to early stages of processing in the visual cortex instead of earlier stages of the CNNs. How does the mapping of earlier stages (e.g., block 1/2) compare to their CNNs trained on 2D tasks? Also it would be good to see a correlation not just between the CNNs at different layers but also between layers. For example, is the correlation between early and late stages in the 2D-trained models higher than between models trained on 3D or semantic tasks?*

We thank the reviewer for the opportunity to clarify our analysis rationale. A consistent choice of layers across tasks was necessary to ensure that differences in results occur only due to different functions DNNs were optimized to perform. Mapping different layers for different functions would violate this requirement. Therefore, we selected the most task-specific layers to represent a given DNN function. We have added a detailed explanation to the manuscript (see excerpts from revised supplementary materials below) why adding early layers which show high correlation between all types of functions (2D, 3D, and semantic) is not suitable if we wish to find the differences between DNN functions.

**Rebuttal Figure 3 (SFigure 2 in revised manuscript supplementary):** **Selecting task-specific DNN representation to compare with fMRI data**: **a)** Spearman’s correlation of all DNN RDMs at a given layer of the encoder with other DNN RDMs computed at the same layer. We report the mean pairwise correlation of all 18 DNNs at different layers of the encoder. **b)** Spearman’s correlation of all DNN RDMs at a given layer of the encoder with a randomly initialized model with the same architecture computed at the same layer. We report the mean correlation of all 18 DNNs with the randomly initialized DNN at different layers of the encoder. **c)** Spearman’s correlation of all DNN RDMs at a given layer of the encoder with deeper layers (block4 and encoder output) of 2D DNNs. We report the mean correlation of the key layers of all 18 DNNs with deeper layers (block4 and encoder output) of 2D DNNs. **d)** Spearman’s correlation between layers at different depths for DNNs corresponding to different task types. We report the mean correlation between different layers averaged across different DNNs of the same task type. **e)** Effect of adding all the key layers on unique and shared variance of fMRI RDMs from different ROIs as compared to selecting only task-specific layers for variance partitioning analysis. We report the change in variance explained (variance change) for 7 variance partitions when all key layers were used for analysis as compared to selecting task-specific layers.

**Excerpts from revised manuscript supplementary section 2**

*“An arguably attractive procedure for layer selection is to select all key layers for each of the DNNs and then perform the comparison. We argue against this by performing an analysis comparing the representation of late layers of 2D DNNs (block 4 and encoder output) with key layers of all the DNNs (Sfigure 2c). We find that early layers of all the DNNs show a high correlation with late layers of 2D DNNs, suggesting that early layers of all DNNs learn a representation required to perform low-level 2D tasks irrespective of the tasks they need to perform (3D or semantic). We further validate this argument by comparing the correlation between different layers of DNNs within a task type (Sfigure 2d). We find that in 2D DNNs the late layers show a high correlation with early layers, suggesting that to perform 2D functions DNNs learn very similar representations at different depths of the network. In the case of 3D and semantic DNNs, the late layers show low correlation with early layers, suggesting that a different representation is required to perform these tasks and that these representations are found in late layers.*

*The early layer representations of all DNNs are very similar to representations learned by 2D DNNs. Including these layers into the variance partitioning analysis could diminish the unique variance of fMRI RDMs explained by 2D DNNs due to an increase in shared variance explained by all the DNNs together. We show the above effect by reporting the change in unique and shared variance when all key layers were used in variance partitioning analysis corresponding to Section 3 of main text instead of the last 2 layers of the encoder (Sfigure 2e). We observe that adding early layers of all 3 different types of DNNs in the analysis leads to an increase in shared variance explained by all these models together and reducing the unique variance contribution of 2D DNNs significantly in the early visual regions. We further observe that in high-level ROIs for which the unique variance of 2D DNNs was insignificant in the original analysis, we barely notice any changes in the unique variance explained. Therefore, to observe the differences in the DNNs due to the task they were optimized to perform we selected the last two layers of the DNNs as the task-specific representation.”*

*Minor comments*

*1) It would be useful to add a figure in the manuscript showing the locations of the cortical areas investigated on an inflated or flattened cortex.*

We thank the reviewer for the suggestion. We have updated Figure 2a in the manuscript to show the location of cortical regions and added an interactive figure on the project website. Please find the figure (Figure2a) below for reference.

**Rebuttal Figure 4 (Figure 2 in revised manuscript):** **Nature and predictive power of the functional map**: **a)** Cortical overlay showing locations of selected cortical regions from the probabilistic atlas used.

*2) It was not clear to me how the representations from the last two layers in the encoder were build. Were the features concatenated? Or averaged? Please provide more details in the methods.*

First we extracted the features for the last two layers in the encoder for all the images in the stimulus set. We then created an RDM for each of these two layers separately, and used those two RDMs as independent variables in regression analysis to predict a given fMRI ROI/searchlight RDM. We have clarified this in the results and methods section. Please find the excerpt from the revised manuscript below.

**Excerpts from revised manuscript Page 20, Lines 613 to 621**

*“We mapped the fMRI responses and DNN activations to corresponding representational dissimilarity matrices (RDMs) by computing pairwise distances between each pair of conditions. We used the variance of upper triangular fMRI RDM (R^2^) explained by DNN RDMs as the measure to quantify the similarity between fMRI responses and DNN activations. To calculate R^2^, we assigned DNN RDMs (two RDMs corresponding to the last two layers of the encoder) as the independent variables and assigned fMRI RDM as the dependent variable. Then a multiple linear regression was fitted to predict fMRI RDM from weighted linear combination of DNN RDMs. We evaluated the fit by estimating the variance explained (R^2^).”*

***Reviewer #2:***

*The authors provide a proof of principle that it is possible to use DNNs, trained on different tasks, to probe the function of brain areas. Using a collection of 18 DNNs, each trained with a different objective / loss function they show that three clusters of DNNs; one related to 2D perception, one related to 3D perception and one related to semantic information map on the expected regions; early visual cortex, the beginning of the dorsal pathway and the beginning of the ventral pathway.*

*That they findings from applying this approach are not surprising is a good thing because it validates the methods. The lack of clear additional findings, beyond the pale of what is roughly known of visual cortex is, from this perspective not a problem.*

*This is also a really nice illustration of the possibilities that the open science movement is providing because the paper combined the neural networks from one publication with the MRI data form another.*

*This is a novel approach and exciting approach. I do think that elements of the paper are overstated and some additional analysis might be sensible.*

We thank the reviewer for considering our approach novel and exciting. We address the reviewer's concern in detail below.

*• This is one novel approach of mapping computational function to brain areas. It would be sensible to place this in a broader context of modern mapping research (for instance, researchers using genetic algorithms to maximally explain responses, or that use brain activity to train DNNs, for instance Seeliger et al, 2021). Given that the main novelty of this paper is in its approach it is sensible to discuss this in a broader framework.*

Thanks for the suggestion. We have added a paragraph discussing our work in a broader context. Please find the excerpt from the revised manuscript below.

**Excerpts from revised manuscript Page 16, Lines 496 to 507**

*“Our study is related to a group of studies^53-55,61^ applying DNNs in different ways to achieve a similar goal of mapping functions of brain regions using DNNs. Some studies^53-54,61^ applied optimization algorithms (genetic algorithm or activation maximization) to find images that maximally activate a given neuron’s or group of neurons' response. Another related study^55^ proposes Neural Information Flow (NIF) to investigate functions of brain regions where they train a DNN with the objective function to predict brain activity while preserving a one-to-one correspondence between DNN layers and biological neural populations. While sharing the overall goal to discover functions of brain regions, investigating DNN functions allows investigation in terms of which computational goal a given brain region is best aligned with. With new computer vision datasets^62^ investigating a diverse set of tasks relevant to human behavioral goals^63,64^ our approach opens new avenues to investigate brain functions.*

*53. Ponce, C.R., Xiao, W., Schade, P.F., Hartmann, T.S., Kreiman, G. and Livingstone, M.S., 2019. Evolving images for visual neurons using a deep generative network reveals coding principles and neuronal preferences. Cell, 177(4), pp.999-1009.*

*54. Bashivan, P., Kar, K. and DiCarlo, J.J., 2019. Neural population control via deep image synthesis. Science, 364(6439).*

*55. Seeliger, K., Ambrogioni, L., Güçlütürk, Y., van den Bulk, L.M., Güçlü, U. and van Gerven, M.A.J., 2021. End-to-end neural system identification with neural information flow. PLoS Computational Biology, 17(2), p.e1008558.*

*61. Gu, Z., Jamison, K.W., Khosla, M., Allen, E.J., Wu, Y., Naselaris, T., Kay, K., Sabuncu, M.R. and Kuceyeski, A., 2021. NeuroGen: activation optimized image synthesis for discovery neuroscience. arXiv preprint arXiv:2105.07140.”*

*• From another perspective there are also approaches in which a network is trained with multiple loss functions at the same time (for instance the UberNet of Kokkinos et al, 2016). This is from a different perspective another approach for mapping function to cortex. Again given that the main novelty of this paper is in its approach it is sensible to discuss this in a broader framework*

Thanks for the suggestion. We have added a paragraph discussing our work in a broader context. Please find excerpt from the revised manuscript below

**Excerpts from revised manuscript Page 17, Lines 521 to 525**

*“While in this study we considered DNNs that were trained independently, future studies might consider investigating multitask models^56,57^ which are trained to perform a wide range of functions using a single DNN. Multitask modeling has the potential to model the entire visual cortex using a single model as compared to several independent models used in this study.*

*56. Scholte, H.S., Losch, M.M., Ramakrishnan, K., de Haan, E.H. and Bohte, S.M., 2018. Visual pathways from the perspective of cost functions and multi-task deep neural networks. cortex, 98, pp.249-261.*

*57. Kokkinos, I., 2017. Ubernet: Training a universal convolutional neural network for low-, mid-, and high-level vision using diverse datasets and limited memory. In Proceedings of the IEEE Conference on Computer Vision and Pattern Recognition (pp. 6129-6138).”*

*• Given that the network was trained on a different data-set than the data-set used for MRI it is important to describe its relationship. In terms of absolute R2 2D loss function DNNs are much better at explaining variance the than 3D / semantic loss function trained DNNs. Likely this is only due to the BOLD-MRI data-quality, but it would be good to hear that the variance in the MRI -dataset is not biased towards the 2D functions (and even if it is this is not a problem for this point of principle study, but still good to know).*

We agree that in terms of absolute R^2^ 2D DNNs are much better than 3D/semantic DNNs for early visual areas. We argue however that this is not the case beyond early visual areas.To demonstrate that the variance in fMRI dataset is not strongly biased towards 2D DNNs we looked at the absolute R^2^ for all tasks separately in functionally localized ROIs (Rebuttal Figure 5; Supplementary SFigure 3) and atlas ROIs (Rebuttal Figure 6; Supplementary SFigure 4). We observe that in OPA 3D DNNs are the best and PPA semantic DNNs are the best. A similar trend can be found in V3d, LO1, LO2, V3a and V3b where 3D DNNs are the best, and VO1, VO2, and PHC1 where semantic DNNs are the best. We have added these figures also in the supplementary material.

**Rebuttal Figure 5 (SFigure 3 in revised manuscript supplementary):** **R^2^ ranking for 18 Taskonomy DNNs and random baseline in functionally localized ROIs.** The bar plot shows the absolute total variance of each ROI RDM explained by task-specific layer RDMs of a given DNNs. The asterisk denotes the significance of total variance (p<0.05, permutation test with 10,000 iterations, FDR-corrected across DNNs). The error bars show the standard deviation calculated by bootstrapping 90% of the conditions (10,000 iterations).

**Rebuttal Figure 6 (SFigure 4 in revised manuscript supplementary):** **R^2^ ranking for 18 Taskonomy DNNs and random baseline in anatomical ROIs.** The bar plot shows the absolute total variance of each ROI RDM explained by task-specific layer RDMs of a given DNNs. The asterisk denotes the significance of total variance (p<0.05, permutation test with 10,000 iterations, FDR-corrected across DNNs). The error bars show the standard deviation calculated by bootstrapping 90% of the conditions (10,000 iterations).

*• Looking at figure 2 it is clear that the noise level for areas beyond V3 (or even beyond V1) are low. This would suggest (see the preceding point) that the data-set is limited in terms of its variance. I have seen datasets where the noise ceiling drops less dramaticly between V1 and V3. Is this problematic for the analysis? At least I find it problematic that the researchers say they explain 60% of the explainable variance without pointing out that the data for these areas is very very noisy (and so without reporting the absolute r2).*

Thank you for the suggestion. We have added R^2^ values observed across these 15 ROIs in the main text. Please find the excerpt from the revised manuscript below.

**Excerpts from revised manuscript Page 9, Lines 260 to 266**

*“We found that variance explained in nine ROIs (V1v, V1d, V2v, V3v, VO1, PHC1, LO2, LO1, V3a) is higher than 60% of the lower bound of noise ceiling (Figure 2c, absolute R^2^ = 0.085 ± 0.046). In absolute terms, the minimum, median and maximum cross-validated R^2^ values across the 15 ROIs were 0.014 (PHC2), 0.044 (VO1) and 0.27 (V1v) which are comparable to related studies^60^ performing evaluation in a similar manner. This shows that the DNNs selected in this study predict fMRI responses well and therefore are suitable for mapping the functions of the investigated ROIs. ”*

We would further like to point out that noise ceiling reported in papers related to ours (e.g. Groen et al. 2018) is usually in terms of R, not R^2^ as here. Therefore when looking at R values the drop the reviewer observes looks less dramatic (Reviewer 1 had a similar concern). We have reproduced the figure with R values (Rebuttal Figure 2) below for reference.

**Rebuttal Figure 2 (not included in manuscript):** **Comparison to noise ceiling (R)**: Pearson’s correlation (R) between fitted RDM using linear combination of top-3 best predicting DNN RDMs (cross validated across subjects and conditions) and ROI RDM indicated in blue bars compared with lower and upper bound of noise ceiling indicated by shaded gray region. The error bars show the 95% confidence interval calculated across N=16 subjects. All the R values are statistically significant (p<0.05, two-sided t-test, FDR-corrected across ROIs).

*• The researchers observe that the earlier layers are more correlated with each other and the later layers are more function specific. They therefore select the final 2 layers of the encoding network. However, given the many studies find the early visual cortex can be well explained by early layers of DNNs it would be sensible to include the first 2 layers as a baseline condition. I would expect that a part of the 2D responses are better explained by the first 2 layers, than be by the last two layers. Likely a (part of) V1 would fall out of analysis. Alternatively, even V1 is more complex than other analysis suggest.*

We agree with the reviewer that the early visual cortex can be well explained by early layers of DNNs and that is what we found in our analysis, too. However, adding early layers of DNNs as a baseline slightly deviates from the key goal of this paper (R1 made a similar suggestion). All the DNNs irrespective of the functions they were performing (2D, 3D, or semantic) showed representation similar to representation learned by deeper layers of 2D DNNs (Rebuttal Figure 3c; SFigure 2c in the revised supplementary material). If our goal had been to determine whether 3D or semantic DNNs learn 2D representations in early layers, then adding early layers would have been a suitable choice. However, in this study our goal was different: we wanted to apply task-specific representation learned by different DNNs to discover which brain regions are better explained by which task-specific representation.

If we take early layers into account, the contribution of 2D DNNs in unique variance is reduced, resulting in higher shared variance among 3 task types (Rebuttal Figure 3e; SFigure 2e in the revised supplementary material). This would lead to the interpretation that 2D DNNs explain less unique variance in early visual areas. We believe this interpretation would be misguided. Instead, the reason is that the 3D DNN and the semantic DNNs have 2D-like representations in their early layers, shifting the contribution of 2D DNNs to shared variance. Please find below the excerpt from the revised supplementary where we added the above discussion to clarify our analysis choices.

**Rebuttal Figure 3 (SFigure 2 in revised manuscript supplementary):** **Selecting task-specific DNN representation to compare with fMRI data**: **a)** Spearman’s correlation of all DNN RDMs at a given layer of the encoder with other DNN RDMs computed at the same layer. We report the mean pairwise correlation of all 18 DNNs at different layers of the encoder. **b)** Spearman’s correlation of all DNN RDMs at a given layer of the encoder with a randomly initialized model with the same architecture computed at the same layer. We report the mean correlation of all 18 DNNs with the randomly initialized DNN at different layers of the encoder. **c)** Spearman’s correlation of all DNN RDMs at a given layer of the encoder with deeper layers (block4 and encoder output) of 2D DNNs. We report the mean correlation of the key layers of all 18 DNNs with deeper layers (block4 and encoder output) of 2D DNNs. **d)** Spearman’s correlation between layers at different depths for DNNs corresponding to different task types. We report the mean correlation between different layers averaged across different DNNs of the same task type. **e)** Effect of adding all the key layers on unique and shared variance of fMRI RDMs from different ROIs as compared to selecting only task-specific layers for variance partitioning analysis. We report the change in variance explained (variance change) for 7 variance partitions when all key layers were used for analysis as compared to selecting task-specific layers.

**Excerpts from revised manuscript supplementary section 2**

*“An arguably attractive procedure for layer selection is to select all key layers for each of the DNNs and then perform the comparison. We argue against this by performing an analysis comparing the representation of late layers of 2D DNNs (block 4 and encoder output) with key layers of all the DNNs (Sfigure 2c). We find that early layers of all the DNNs show a high correlation with late layers of 2D DNNs, suggesting that early layers of all DNNs learn a representation required to perform low-level 2D tasks irrespective of the tasks they need to perform (3D or semantic). We further validate this argument by comparing the correlation between different layers of DNNs within a task type (Sfigure 2d). We find that in 2D DNNs the late layers show a high correlation with early layers, suggesting that to perform 2D functions DNNs learn very similar representations at different depths of the network. In the case of 3D and semantic DNNs, the late layers show low correlation with early layers, suggesting that a different representation is required to perform these tasks and that these representations are found in late layers.*

*The early layer representations of all DNNs are very similar to representations learned by 2D DNNs. Including these layers into the variance partitioning analysis could diminish the unique variance of fMRI RDMs explained by 2D DNNs due to an increase in shared variance explained by all the DNNs together. We show the above effect by reporting the change in unique and shared variance when all key layers were used in variance partitioning analysis corresponding to Section 3 of main text instead of the last 2 layers of the encoder (Sfigure 2e). We observe that adding early layers of all 3 different types of DNNs in the analysis leads to an increase in shared variance explained by all these models together and reducing the unique variance contribution of 2D DNNs significantly in the early visual regions. We further observe that in high-level ROIs for which the unique variance of 2D DNNs was insignificant in the original analysis, we barely notice any changes in the unique variance explained. Therefore, to observe the differences in the DNNs due to the task they were optimized to perform we selected the last two layers of the DNNs as the task-specific representation.”*

*• The researchers select from the 2D networks (7 loss functions) and 3D networks (8 loss functions) the 3 best performing networks. This is not possible with the semantic networks (only 3 loss functions come into play). Again, this is not a problem for the point of principle approach but is can result in effects on the r2 that are an effect of the selection procedure (vs the function. To unpack that, if loss functions are correlated they will share more units, and are therefore less capable of explaining variance than if they are uncorrelated (for instance see Scholte et al., 2018). Therefore, if the correlations between the loss functions are greater for the 2D functions (or 3D functions) they would be biased to have a higher r2. Vice versa, it is a relief to see that the semantic network is winning in VO1/VO2. Again this is not a problem for the point of principle nature of the paper but more on the correlation structure of the loss would be informative to interpret the results.*

This is an important point to consider. First, we would like to clarify that the top-3 best performing networks were selected from the set of 18 DNNs (and not from the individual group), so the selection procedure is unbiased with respect to grouping. However, we agree that if the differences in the R^2^ values across DNNs were not significant, the selection process would be biased towards 2D and 3D DNNs which are more numerous than semantic DNNs.

We further agree that the correlation structure plays a role in variance partitioning analysis. In the current version of the manuscript we clarify the role of the correlation structure in the results section to avoid misinterpretation of the results. Please find the excerpt from the revised manuscript below.

**Excerpts from revised manuscript Page 9, Lines 226 to 235**

*“It is crucial to note that if two DNNs from the same task group are in the top-3 best predicting DNNs for a ROI, the unique variance of ROI RDM explained by DNNs in the same group will generally be lower than by DNN not in the group. We have observed that DNNs in the same task group show a higher correlation with each other as compared to DNNs in other task groups^14^. A higher correlation between the DNNs of the same task group leads to an increase in shared variance and reduces the unique variance of the ROI RDM explained by within task group DNNs. For instance, we can observe this in PHC2 (also in PHC1, V3a), where two semantic DNNs explain less unique variance than a 3D DNN. Therefore, in such cases we restrain from interpreting that one type of DNN is significantly better than others.”*

To tackle the biased selection and correlation structure issue we performed variance partitioning analysis at grouped DNN level in Results section 3 (Figure 3) of the manuscript. Grouped DNN RDMs for each task type were obtained by averaging the RDMs within a task group (e.g. 2D) resulting in an equal number of RDMs corresponding to 2D, 3D and semantic DNNs. Therefore, in the grouped DNN level we mitigate the selection bias mentioned by the reviewer.

*• It is not entirely clear to me what the novel functional insights for the 15 ROIs are. If they are really in the paper it would be sensible to outline this more directly.*

Thank you for giving us the opportunity to clarify. We have outlined these insights in the discussion more clearly. Please find the excerpt below.

**Excerpts from revised manuscript Page 14-16, Lines 418 to 471**

*“We obtained the functional mapping of different regions in the visual cortex on both the individual (e.g., 2D edges, scene classification, surface normals, etc.) and the group (2D, 3D, semantic) level of visual functions. We discuss the novel insights gained at the level of individual functions that inform about the fine-grained functional role of cortical regions.*

*First we consider 2D DNNs, where the denoising DNN explained significant unique variance in V1v, V1d, V2v, V2d, V3v and hV4. The denoising task requires the DNN to reconstruct an unperturbed input image from slight perturbed (e.g. adding Gaussian noise in the current case) in the input image that encourages learning representations robust to slight perturbations and limited invariance. This suggests that these ROIs might be generating a scene representation robust to high frequency noise.*

*When considering 3D DNNs, the 3D Keypoint and the 2.5d segment were among top-3 best predicting DNNs in multiple ROIs. The 3D Keypoints DNN explained significant unique variance in V3d, PHC1, PHC2, LO2, LO1, V3a, V3b, OPA and PPA. The 3D keypoints task requires the DNN to identify locally important regions of the input image based on object boundary information and surface stability. This suggests that the ROIs in which 3D Keypoints DNN explained significant variance may be identifying locally important regions in a scene. The identification of locally important regions might be relevant to selectively attend to these key regions to achieve a behavioral goal e.g. searching for an object. The 2.5d segment DNN explained significant unique variance in V3d, LO2, LO1, V3b, V3a and OPA. The 2.5d segment task requires the DNN to segment images into perceptually similar groups based on color and scene geometry (depth and surface normals). This suggests that the ROIs in which 2.5d segment DNN explained significant variance may be grouping regions in the images based on color and geometry cues even without any knowledge of the categorical information. Grouping regions based on geometry could be relevant to behavioral goals such as reaching for objects or identifying obstacles.*

*Among semantic DNNs, the semantic segmentation DNN explained significant unique variance in VO1, VO2, PHC1, PHC2, V3a and PPA. The semantic segmentation task requires the DNN to segment objects present in the image based on categories. This suggests that the ROIs in which semantic segmentation DNN explained significant variance may be grouping regions in the image based on categorical information.*

*Other DNNs (2D edges, scene classification, and object classification) that showed significant unique variance in ROIs provided functional insights mostly consistent with the previous studies^23–25,31,32^. Overall, the key DNNs (denoising, 3D keypoints , 2.5D segment and semantic segmentation) that explained significant variance in multiple ROI responses uniquely promote further investigation by generating novel hypotheses about the functions of these ROIs. Future experiments can test these hypotheses in detail in dedicated experiments.*

*The functional mapping obtained using grouped DNNs is complementary to that at the individual level, and helps us put functional mapping obtained here in context with previous literature. We found that early visual regions (V1v, V1d, V2v) have a functional role related to low-level 2D visual tasks which is consistent with previous literature investigating these regions^23–25^. In dorsal-ventral ROIs (V3a, V3d, LO1, and LO2) we found functional roles related to 3D and semantic tasks converging with evidence from previous studies^26–30^. Similarly, the prominent semantic functional role of later ventral-temporal ROIs (VO1, VO2, PHC1, and PHC2) found in this study converges with findings in previous literature^31,32^. In scene-selective ROIs, we found a semantic functional role for PPA and 3D functional role for OPA respectively. Our study extends the findings of a previous study^51^ relating OPA and PPA to 3D models by differentiating between OPA and PPA functions through a much broader set of models. To summarize, the functional mapping using individual DNNs optimized to perform different functions revealed new functional insights for higher ROIs in the visual cortex while at the same time functional mapping using grouped DNNs showed highly converging evidence with previous independent studies investigating these ROIs.”*

*• The authors take a stance on cognitive ontology. They refer to the framework of Marr in which function exists next to computation / implementation. However, an alternative is that there is a many to one, or even many to many mapping (see for instance: Francken, J. C., & Slors, M. (2014). From commonsense to science, and back: The use of cognitive concepts in neuroscience. Consciousness and cognition, 29, 248-258 or Klein, C. (2012). Cognitive ontology and region-versus network-oriented analyses. Philosophy of Science, 79(5), 952-960). Feel free to ignore the reference but using DNNs in this manner is sensible (or even more sensible) from this perspective.*

We have updated the discussion to include alternative hypotheses as suggested by the reviewer. In fact, we did find many-to-one mappings in several ROIs suggesting 3D + semantic functional roles. Please find the relevant excerpt from the revised manuscript below.

**Excerpts from revised manuscript Page 16, Lines 486 to 492**

*“Our high-throughput exploration of potential computational functions was initially inspired by Marr's computational level of analysis^44^ which aims at finding out what the goal of the computation carried out by a brain region is. While Marr’s approach invites the expectation of a one-to-one mapping between regions and goals, we found evidence for multiple functional roles (3D + semantic) using DNNs in some ROIs (e.g. LO1, LO2, PHC1, PHC2). This indicates a many-to-one mapping^59^ between functions and brain regions.”*

***Reviewer #3:***

*In this manuscript, fMRI responses to 50 images of indoor scenes are compared against feature activations to these images in upper layers of a large number of DNNs obtained from the Taskonomy project, whereby each DNN was trained on a different task (with the same architecture). The ability of these DNNs to predict fMRI responses is evaluated via representational similarity analysis, in both a set of ROIs defined from a retinotopic atlas (visual regions V1 up to VO1/2 ventral and IPS) and in a search through the entire recorded brain volume. The authors find that DNNs pre-trained on different tasks (but with otherwise identical architectures) best predict different brain regions. In particular, representational similarity in lower visual regions is best predicted by DNNs trained on ‘low-level’ tasks like edge detection, ventral stream regions are best predicted by semantic tasks, and dorsal stream regions are best predicted by tasks that require representing 3D structure (and also semantic tasks). The authors conclude that the study ‘provides a comprehensive account of the function of human visual brain regions.’*

*Overall, I am sympathetic to the author’s goals here: I think the idea here is good and relevant, and I agree that this study is probably the largest so far in systematically comparing the effect that the task DNNs are trained on has on their ability to map fMRI responses in visual cortex. I also think that overall, the analyses are well executed (e.g., using variance partitioning on both the individual tasks and the task groups, with nice 3D visualizations), perhaps with the exception of the calculations going into the comparison of model explained variance with the maximal explainable variance, which may warrant some more substantiation (see comments below).*

We thank the reviewer for considering our analysis well executed. We have addressed the reviewer’s concern about comparison of model explained variance with the maximal explainable variance below in their specific comment related to that point.

*However, there are some weaknesses too. In particular, the claim above (“a comprehensive account”) is a bit of an overstatement, especially since very little interpretation is in fact offered of the obtained mappings. As pointed out by the authors, the mapping they find between the task groups and visual cortical regions ‘converges with’ a lot of prior research assigning functions to visual regions; it is indeed entirely consistent with our now a few decades old ‘standard’ model of visual processing existing of an early visual stage followed by a ventral/dorsal what/where pathway. So, a question that emerges is ‘do we actually learn something new here about how visual cortex is organized’? In this sense, the more fine-grained mapping of the individual tasks to individual ROIs offers potentially more interesting novel insights. However, I found these mappings somewhat difficult to make sense of, for two reasons: 1) results are obtained for one dataset with 50 images only, so may be idiosyncratic to the dataset and 2) there is very little explanation about the nature of the different tasks that are compared, and whether they may resemble tasks that the human visual system is also trying to solve.*

We thank the reviewer for providing us the opportunity to clarify which findings converge with prior research and which findings are novel. We have explained the nature of the tasks for the key DNNs that best predicted fMRI responses and their potential relevance to the human visual system in the updated manuscript. We agree with the reviewer about the limitation due to the fMRI dataset and have discussed this in the revised version. Please find details in our responses to specific comments below.

*Below, I provide some more detailed comments and suggestions that I hope will help the authors improve their manuscript.*

*MAIN COMMENTS*

*Methods:*

*As mentioned above, one limitation of this study is the fact that only one fMRI dataset with a relatively small set of images is tested, leaving open the question about to what extent the mapping of e.g., the ‘3D keypoints’ task with area V3b is a ‘real’ consistent finding or have something to do with the particular images used here. I realize it may not be easy to find another high-quality fMRI data set that uses indoor scenes (which the Taskonomy DNNs are optimized for), but (also given that the authors have themselves already published several papers on these data), I encourage them to consider testing the same models against another dataset, which I believe would greatly strengthen the paper (if it indeed shows consistent results as reported here). For example, perhaps there is a subset of images in the BOLD5000 dataset that are suitable for this purpose?*

We agree with the reviewer that replicating the results on a separate fMRI dataset would greatly strengthen the paper. However, the BOLD5000 dataset is unfortunately not well suited for this purpose. BOLD5000 has a much broader sampling of images, but at the cost of lower SNR due to less number of trials (1-4) per condition as compared to the data we used here (10 trials per condition). The effect is reflected in correlation between brain and model RDMs (maximal correlations to a DNN of 0.16 in BOLD versus correlations of >0.5 found for the data set used here). This suggests that the noise ceiling of the BOLD5000 dataset will be lower and that it does not afford the power for extensive testing of model comparisons of the type that we perform here.

We discuss this limitation in the current version of the manuscript.

**Excerpts from revised manuscript Page 17, Lines 525 to 527**

*“Another potential limitation is that our findings are based on a single fMRI and image dataset, so that it is not clear how well they would generalize to a broader sample of images.”*

*It would be really helpful if the paper would provide more insight into the nature of the different Taskonomy tasks, and if and how we should think of them as being ‘valid’ model tasks for what the brain might be doing. For example, autoencoding or denoising may not really be akin to the brain’s computational goals, whereas 2D edges may in fact be very much like the computational goal of, say, V1. I’m not saying the authors have to make strong claims, but it could be interesting to use these results to provide some insight into this question: what are ‘plausible’ functions of the brain regions studied here?*

We thank the reviewer for the opportunity to clarify (Reviewer 2 had a similar concern). We have updated the discussion section to describe the tasks of key DNNs that explained unique variance in multiple ROIs significantly along with potential hypotheses about how these tasks might be relevant to the brain's computation goal. Please find the excerpt from the revised manuscript below.

**Excerpts from revised manuscript Page 14-15, Lines 422 to 455**

*“First we consider 2D DNNs, where the denoising DNN explained significant unique variance in V1v, V1d, V2v, V2d, V3v and hV4. The denoising task requires the DNN to reconstruct an unperturbed input image from slight perturbed (e.g. adding Gaussian noise in the current case) in the input image that encourages learning representations robust to slight perturbations and limited invariance. This suggests that these ROIs might be generating a scene representation robust to high frequency noise.*

*When considering 3D DNNs, the 3D Keypoint and the 2.5d segment were among top-3 best predicting DNNs in multiple ROIs. The 3D Keypoints DNN explained significant unique variance in V3d, PHC1, PHC2, LO2, LO1, V3a, V3b, OPA and PPA. The 3D keypoints task requires the DNN to identify locally important regions of the input image based on object boundary information and surface stability. This suggests that the ROIs in which 3D Keypoints DNN explained significant variance may be identifying locally important regions in a scene. The identification of locally important regions might be relevant to selectively attend to these key regions to achieve a behavioral goal e.g. searching for an object. The 2.5d segment DNN explained significant unique variance in V3d, LO2, LO1, V3b, V3a and OPA. The 2.5d segment task requires the DNN to segment images into perceptually similar groups based on color and scene geometry (depth and surface normals). This suggests that the ROIs in which 2.5d segment DNN explained significant variance may be grouping regions in the images based on color and geometry cues even without any knowledge of the categorical information. Grouping regions based on geometry could be relevant to behavioral goals such as reaching for objects or identifying obstacles.*

*Among semantic DNNs, the semantic segmentation DNN explained significant unique variance in VO1, VO2, PHC1, PHC2, V3a and PPA. The semantic segmentation task requires the DNN to segment objects present in the image based on categories. This suggests that the ROIs in which semantic segmentation DNN explained significant variance may be grouping regions in the image based on categorical information.*

*Other DNNs (2D edges, scene classification, and object classification) that showed significant unique variance in ROIs provided functional insights mostly consistent with the previous studies^23–25,31,32^. Overall, the key DNNs (denoising, 3D keypoints , 2.5D segment and semantic segmentation) that explained significant variance in multiple ROI responses uniquely promote further investigation by generating novel hypotheses about the functions of these ROIs. Future experiments can test these hypotheses in detail in dedicated experiments.”*

*The claim that the models explain up to 60% of the explainable variance is impressive, especially given the fact that RSA studies typically report quite low correlations between model RDMs and fMRI RDMs. From section 4.6, I understand that the noise ceiling computation is an adaptation of the method described in the RSA toolbox paper (Nili et al, 2014) using explained variance rather than correlation. This makes sense to me. But it’s not clear to me that the calculation of the variance explained by the best predicting DNN here is valid in comparison; usually, the (group average of) single-subject correlations between model RDMs and fMRI RDMs are compared against the noise ceiling, but here these numbers are estimated by first fitting model RDMs to a subject-averaged RDM (of N-1 subject) and then testing those fitted regression weights against the RDM of the Nth subject (i.e., leave one out cross-validation). Isn’t this guaranteed to yield much higher R2 values (since you’re using the average to fit)? And how much does the 2N fold cross-validating across conditions affect the estimates, why is that included? In summary, I think that this new method needs to be explained, motivated and validated better, possibly through simulations using ground-truth RDMs, and/or how it compares to the ‘traditional’ RSA noise ceiling comparison using single-subject correlations only.*

To take into account multiple layers in the model we perform regression to predict fMRI RDM (dependent variable) from multiple DNN RDMs (i.e., the 2 RDMs for the last 2 layers of the encoder) as the independent variable. It can be shown that if we have enough independent variables (e.g. 1,000 RDMs) even random RDMs can predict fMRI RDM well (Rebuttal Figure 7b; SFigure 5b in supplementary). Therefore we need to cross-validate using different sets of RDMs for fitting and evaluating the fit of the regression. Here, we perform double cross-validation on subjects and images simultaneously: we fit the regression on a one-subject-left-out (subject-averaged) RDM on half of the images in the stimulus set and evaluate it on the single (left out) subject RDM on the other half of the images. Please note that the above method is a stricter evaluation criteria as compared to the one used in the RSA toolbox version which does not employ cross-validation. Below (Rebuttal Figure 7a; SFigure 5a in supplementary) we show the comparison between the R^2^ using single subject correlations compared with R^2^ using cross validation across subjects and cross validation across both subjects and stimuli. We observe that using cross validation across subjects and stimuli generally yields lower R^2^ values as compared to the traditional method and intersubject cross-validation. The difference is clearer in the case of 1,000 random RDMs (Rebuttal Figure 7b; SFigure 5b in supplementary). Therefore we believe it is a stricter criteria for evaluation. We have motivated the method in the results section and added the figure in the supplementary section. Please find the excerpt from the revised manuscript below.

**Rebuttal Figure 7 (SFigure 5 in revised manuscript supplementary):** **Effect of cross validation on variance explained (R^2^)** **a)** Variance of each ROI explained by top-3 best predicting DNNs compared for different cross-validation settings (blue bars: no cross validation; orange bars: cross validation across subjects; green bars: cross validation across subjects and stimuli). The error bars show the 95% confidence interval calculated across N=16 subjects. All R^2^ values are statistically significant (p<0.05, two-sided t-test, FDR-corrected across ROIs) **b)** Variance of each ROI explained by 1,000 randomly generated RDMs compared for different cross-validation settings (blue bars: no cross validation; orange bars: cross validation across subjects; green bars: cross validation across subjects and stimuli). The error bars show the 95% confidence interval calculated across N=16 subjects.

**Excerpts from revised manuscript Page 9, Lines 251 to 258**

*“As we use multiple models together for multiple linear regression, we need to cross-validate using different sets of RDMs for fitting and evaluating the fit of the regression. Here, we perform cross-validation across subjects by fitting the regression on one-subject-left-out subject-averaged RDMs on half of the images in the stimulus set and evaluating on the left-out single subject RDM on the other half of the images. The above method is a stricter evaluation criterion as compared to the commonly used one without cross-validation (See supplementary SFigure 5).”*

*Results:*

*One thing that struck me as odd about the results is that in all the surface brain maps, the magenta ‘blobs’ (reflecting best predictions by semantic task-trained models) were always surrounded by an edge of green (best predictions by 3D task trained models). Why would this be? Could it be an artifact of the analysis?*

We thank the reviewer for pointing the artifact out. We found that while projecting to the surface the nilearn toolbox in default settings interpolates the values in a given sphere. We changed the relevant parameters to set interpolation to zero and updated all the surface projections in the revised manuscript. For reference we show the comparison between old (Rebuttal Figure 8a; with interpolation) and updated (Rebuttal Figure 8b; without interpolation) Figure 3a below (Rebuttal Figure 8).

**Rebuttal Figure 8 (not included in manuscript):** **Comparison to noise ceiling (R)**: **a) (With interpolation)** Functional map of the visual cortex showing the regions where unique variance explained by one DNN group (2D, 3D, or semantic) is significantly higher than the variance explained by the other two DNN groups using default nilearn settings. **b) (Without interpolation)** Functional map of the visual cortex showing the regions where unique variance explained by one DNN group (2D, 3D, or semantic) is significantly higher than the variance explained by the other two DNN groups using no interpolation.

*Another thing that is striking is that in the dorsolateral view of the maps, there appears to be a strong lateralization with high correspondences with semantic-trained DNNs in the right hemisphere but none in the left hemisphere. Again, why would this be?*

We agree that there seems to be a lateralization with 3D functions prevalent in the left hemisphere, and semantic functions in the right hemisphere. While intriguing, we are hesitant to report and interpret this observation for several reasons. For one, we had no a priori hypothesis about lateralization and thus this observation is post-hoc. Further, we tried and failed to put this finding into context of previous literature in a convincing way. While naturally reports of lateralization of diverse cognitive functions abound across the whole brain, we see no sufficient fit between previous findings and ours. We are therefore at a loss of what to make of it. Finally, we considered several ways of testing for this lateralization statistically to potentially support the observation. However, to yield significant effects all of those would likely involve setting some ROI based on the observation made, which would invite circularity. In total we therefore suggest not to mention this observation in the manuscript if the reviewer permits.

Discussion:

*On line 378, it is suggested that this work highlights the feasibility of a ‘systematic approach that finds the functional roles of multiple brain regions […]’ Proof of principle is one thing, but do the results obtained so far generate any new hypotheses or predictions about the function of particular brain regions? Why (not)?*

We agree that the results obtained from the analysis using DNNs grouped into 2D, 3D and semantic RDMs serve as a proof of principle that our approach is valid and consistent with prior findings. However, the results obtained using the individual DNNs (e.g. 3D keypoints, 2.5 D segment, semantic segmentation) that showed significant unique variance in multiple ROIs generate new hypotheses about the potential functions of the corresponding ROIs. We have modified the discussion section to emphasize what new hypotheses these DNNs generate about different ROIs. Please find the relevant excerpt from the revised manuscript below.

**Excerpts from revised manuscript Page 14-16, Lines 418 to 471**

*“We obtained the functional mapping of different regions in the visual cortex on both the individual (e.g., 2D edges, scene classification, surface normals, etc.) and the group (2D, 3D, semantic) level of visual functions. We discuss the novel insights gained at the level of individual functions that inform about the fine-grained functional role of cortical regions.*

*First we consider 2D DNNs, where the denoising DNN explained significant unique variance in V1v, V1d, V2v, V2d, V3v and hV4. The denoising task requires the DNN to reconstruct an unperturbed input image from slight perturbed (e.g. adding Gaussian noise in the current case) in the input image that encourages learning representations robust to slight perturbations and limited invariance. This suggests that these ROIs might be generating a scene representation robust to high frequency noise.*

*When considering 3D DNNs, the 3D Keypoint and the 2.5d segment were among top-3 best predicting DNNs in multiple ROIs. The 3D Keypoints DNN explained significant unique variance in V3d, PHC1, PHC2, LO2, LO1, V3a, V3b, OPA and PPA. The 3D keypoints task requires the DNN to identify locally important regions of the input image based on object boundary information and surface stability. This suggests that the ROIs in which 3D Keypoints DNN explained significant variance may be identifying locally important regions in a scene. The identification of locally important regions might be relevant to selectively attend to these key regions to achieve a behavioral goal e.g. searching for an object. The 2.5d segment DNN explained significant unique variance in V3d, LO2, LO1, V3b, V3a and OPA. The 2.5d segment task requires the DNN to segment images into perceptually similar groups based on color and scene geometry (depth and surface normals). This suggests that the ROIs in which 2.5d segment DNN explained significant variance may be grouping regions in the images based on color and geometry cues even without any knowledge of the categorical information. Grouping regions based on geometry could be relevant to behavioral goals such as reaching for objects or identifying obstacles.*

*Among semantic DNNs, the semantic segmentation DNN explained significant unique variance in VO1, VO2, PHC1, PHC2, V3a and PPA. The semantic segmentation task requires the DNN to segment objects present in the image based on categories. This suggests that the ROIs in which semantic segmentation DNN explained significant variance may be grouping regions in the image based on categorical information.*

*Other DNNs (2D edges, scene classification, and object classification) that showed significant unique variance in ROIs provided functional insights mostly consistent with the previous studies^23–25,31,32^. Overall, the key DNNs (denoising, 3D keypoints , 2.5D segment and semantic segmentation) that explained significant variance in multiple ROI responses uniquely promote further investigation by generating novel hypotheses about the functions of these ROIs. Future experiments can test these hypotheses in detail in dedicated experiments.*

*The functional mapping obtained using grouped DNNs is complementary to that at the individual level, and helps us put functional mapping obtained here in context with previous literature. We found that early visual regions (V1v, V1d, V2v) have a functional role related to low-level 2D visual tasks which is consistent with previous literature investigating these regions^23–25^. In dorsal-ventral ROIs (V3a, V3d, LO1, and LO2) we found functional roles related to 3D and semantic tasks converging with evidence from previous studies^26–30^. Similarly, the prominent semantic functional role of later ventral-temporal ROIs (VO1, VO2, PHC1, and PHC2) found in this study converges with findings in previous literature^31,32^. In scene-selective ROIs, we found a semantic functional role for PPA and 3D functional role for OPA respectively. Our study extends the findings of a previous study^51^ relating OPA and PPA to 3D models by differentiating between OPA and PPA functions through a much broader set of models. To summarize, the functional mapping using individual DNNs optimized to perform different functions revealed new functional insights for higher ROIs in the visual cortex while at the same time functional mapping using grouped DNNs showed highly converging evidence with previous independent studies investigating these ROIs.”*

MINOR COMMENTS

*According to the Methods, feature activations from the top 2 layers of each DNN are extracted and compared to the brain data, but it isn’t clear how these are then used in subsequent analysis. For example, is each layer used in the partitioning analysis, or do you pick the highest correlating one each time? Relatedly, I didn’t really follow the explanation offered on lines 464-469 about why the selection of the 2 top layers ‘ensured that differences in representation were due to the functions DNNs were optimized for’.*

We thank the reviewer for providing us the opportunity to clarify our methods (A similar clarification was requested by Reviewer 1). First we extracted the features for the last two layers in the encoder for all the images in the stimulus set. Then, we created 2 RDMs one corresponding to each layer which were then used as independent variables in regression analysis to predict a given fMRI ROI/searchlight RDM. We have clarified this in the results and methods section. Please find the excerpt from the revised manuscript below.

**Excerpts from revised manuscript Page 20, Lines 613 to 621**

*“We mapped the fMRI responses and DNN activations to corresponding representational dissimilarity matrices (RDMs) by computing pairwise distances between each pair of conditions. We used the variance of upper triangular fMRI RDM (R^2^) explained by DNN RDMs as the measure to quantify the similarity between fMRI responses and DNN activations. To calculate R^2^, we assigned DNN RDMs (RDMs of the last two layers of the encoder) as the independent variables and assigned fMRI RDM as the dependent variable. Then a multiple linear regression was fitted to predict fMRI RDM from weighted linear combination of DNN RDMs. We evaluated the fit by estimating the variance explained (R^2^). ”*

To ensure that differences in representation do not arise due to difference in layer choice between DNNs, we made a consistent layer choice across all the DNNs. For the choice to be fair and representative of the task DNN was optimized for, we have to choose task-specific layers (deeper layers of the encoder) while ignoring the earlier layers that learn a generic representation irrespective of tasks. We have updated the layer selection section in the supplementary in detail (A clarification about layer selection was also requested by Reviewers 1&2). Please find the relevant text and figures below.

**Rebuttal Figure 3 (SFigure 2 in revised manuscript supplementary):** **Selecting task-specific DNN representation to compare with fMRI data**: **a)** Spearman’s correlation of all DNN RDMs at a given layer of the encoder with other DNN RDMs computed at the same layer. We report the mean pairwise correlation of all 18 DNNs at different layers of the encoder. **b)** Spearman’s correlation of all DNN RDMs at a given layer of the encoder with a randomly initialized model with the same architecture computed at the same layer. We report the mean correlation of all 18 DNNs with the randomly initialized DNN at different layers of the encoder. **c)** Spearman’s correlation of all DNN RDMs at a given layer of the encoder with deeper layers (block4 and encoder output) of 2D DNNs. We report the mean correlation of the key layers of all 18 DNNs with deeper layers (block4 and encoder output) of 2D DNNs. **d)** Spearman’s correlation between layers at different depths for DNNs corresponding to different task types. We report the mean correlation between different layers averaged across different DNNs of the same task type. **e)** Effect of adding all the key layers on unique and shared variance of fMRI RDMs from different ROIs as compared to selecting only task-specific layers for variance partitioning analysis. We report the change in variance explained (variance change) for 7 variance partitions when all key layers were used for analysis as compared to selecting task-specific layers.

**Excerpts from revised manuscript supplementary section 2**

*“An arguably attractive procedure for layer selection is to select all key layers for each of the DNNs and then perform the comparison. We argue against this by performing an analysis comparing the representation of late layers of 2D DNNs (block 4 and encoder output) with key layers of all the DNNs (Sfigure 2c). We find that early layers of all the DNNs show a high correlation with late layers of 2D DNNs, suggesting that early layers of all DNNs learn a representation required to perform low-level 2D tasks irrespective of the tasks they need to perform (3D or semantic). We further validate this argument by comparing the correlation between different layers of DNNs within a task type (Sfigure 2d). We find that in 2D DNNs the late layers show a high correlation with early layers, suggesting that to perform 2D functions DNNs learn very similar representations at different depths of the network. In the case of 3D and semantic DNNs, the late layers show low correlation with early layers, suggesting that a different representation is required to perform these tasks and that these representations are found in late layers.*

*The early layer representations of all DNNs are very similar to representations learned by 2D DNNs. Including these layers into the variance partitioning analysis could diminish the unique variance of fMRI RDMs explained by 2D DNNs due to an increase in shared variance explained by all the DNNs together. We show the above effect by reporting the change in unique and shared variance when all key layers were used in variance partitioning analysis corresponding to Section 3 of main text instead of the last 2 layers of the encoder (Sfigure 2e). We observe that adding early layers of all 3 different types of DNNs in the analysis leads to an increase in shared variance explained by all these models together and reducing the unique variance contribution of 2D DNNs significantly in the early visual regions. We further observe that in high-level ROIs for which the unique variance of 2D DNNs was insignificant in the original analysis, we barely notice any changes in the unique variance explained. Therefore, to observe the differences in the DNNs due to the task they were optimized to perform we selected the last two layers of the DNNs as the task-specific representation.”*

*Methods line 573: For the ROI analysis, is the FDR correction applied only across models, or also across ROIs?*

FDR correction is applied only across models as we were investigating each ROI in an independent manner. We clarified this at all appropriate places in the methods and results section.

*For Figure 1d, it is really hard to see any distinction between the blues, or between the different 3D or semantic tasks. In this sense, the figure is almost showing the same thing as Figure 3 where the RDMs are averaged within task groups prior to performing the searchlight. Would it be possible to provide additional maps showing only one task group, but with a more diverging color scale, for example?*

Thanks for the suggestion! We provide additional visualization for each task group with more diverging colors on the project website. Please find the visualization here : <https://sites.google.com/view/dnn2brainfunction/home#h.ga0el97qakmk>

*It could be nice to show a couple of ROIs in more detail, for example how they represent the different tasks within a group (not just the top 3).*

We thank the reviewer for the suggestion (Reviewer 2 had a similar request). We have therefore added the R^2^ plots including all 19 DNNs (18 tasks + random baselines) for all the ROIs in the supplementary material (Supplementary Figure 3 and Figure 4). We have reproduced the figures below for reference.

**Rebuttal Figure 4 (SFigure 3 in revised manuscript supplementary):** **R^2^ ranking for 18 Taskonomy DNNs and random baseline in functionally localized ROIs.** The bar plot shows the absolute total variance of each ROI RDM explained by task-specific layer RDMs of a given DNNs. The asterisk denotes the significance of total variance (p<0.05, permutation test with 10,000 iterations, FDR-corrected across DNNs). The error bars show the standard deviation calculated by bootstrapping 90% of the conditions (10,000 iterations).

**Rebuttal Figure 5 (SFigure 4 in revised manuscript supplementary):** **R^2^ ranking for 18 Taskonomy DNNs and random baseline in anatomical ROIs.** The bar plot shows the absolute total variance of each ROI RDM explained by task-specific layer RDMs of a given DNNs. The asterisk denotes the significance of total variance (p<0.05, permutation test with 10,000 iterations, FDR-corrected across DNNs). The error bars show the standard deviation calculated by bootstrapping 90% of the conditions (10,000 iterations).

*In Supp Section 2: The early layers of different DNNs show more similarity to a random init model then the later layers. Why is this the case? Do lower layers become less tuned than higher layers in training? Are they more shaped by the built-in architecture?*

These are interesting and open questions! We believe a possible explanation of why early layers of different DNNs show more similarity to a randomly initialized model than the later layers is that they require less tuning as compared to deeper layers to perform a specific task. This explanation is supported in a related machine learning study (Raghu et al. 2017) where it was found that the weights update in training occurs in the bottom up manner with early layers converging first and deeper layers converging the last. While this could be a potential reason for what we observe in our study, we prefer not to make any claim without any analysis to support it.

We have no clear answer whether the representation of early layers is shaped by architecture or not. That would require an exhaustive analysis by comparing multiple architectures with untrained and trained models on different tasks. This is however beyond the scope of the manuscript

Although both the analyses investigating the representations of early layers are interesting,we believe that these are not directly relevant to the main question of the current study. Therefore we restrain ourselves from mentioning any specific reason why early layers show more similarity with a random model without the support of exhaustive analyses.

Raghu, M., Gilmer, J., Yosinski, J. and Sohl-Dickstein, J., 2017. Svcca: Singular vector canonical correlation analysis for deep learning dynamics and interpretability. *arXiv preprint arXiv:1706.05806*.

*Discussion:*

*Given the large list of prior findings the results are consistent with, the findings here come across as purely confirmatory (and thus a bit boring). I’m wondering if a stronger claim can be made that this could be a proof of principle of not just that ‘it is possible to map the brain with DNNs’ but that the brain could - in theory - achieve different functions with the same canonical architecture and set of computations. In other words, it may not be necessary to have an entirely different type of CNN (with e.g. spatiotemporal units) to explain dorsal stream regions. Or does that go too far?*

We would be thrilled if we could make the claim that the brain can achieve different functions with the same canonical architecture based on our results. However, we do not believe our work to provide enough evidence for such a strong claim. Here the same architecture was chosen to ensure that the difference in variance explained by different models cannot be attributed to architecture. We strongly believe that CNNs with different architectures will explain different parts of the brain differentially well.

*The authors seem to have missed a few relevant cites:*

*- Guclu & van Gerven (2017, NeuroImage) show a correspondence between dorsal visual regions and a DNN trained on action recognition (effectively also showing that dorsal regions are better explained by a different-trained CNN than object recognition)*

*- Lescroart & Gallant (2019, Neuron) showed that 3D information is a better predictor than 2D information is high-level scene regions through direct model comparison*

Thanks for mentioning this. We have added the citations in the discussion section. Please find the excerpts from the revised manuscript below.

**Excerpts from revised manuscript Page 14, Lines 410 to 412**

*“Previous studies*[*^3,4,6,7,17–21^*](https://www.zotero.org/google-docs/?j3B5Re)*^,51,52^ have compared model performance in explaining brain activity, but were limited to a few preselected regions and models.*

*51. Lescroart, M.D. and Gallant, J.L., 2019. Human scene-selective areas represent 3D configurations of surfaces. Neuron, 101(1), pp.178-192.*

*52. Güçlü, U. and van Gerven, M.A., 2017. Increasingly complex representations of natural movies across the dorsal stream are shared between subjects. NeuroImage, 145, pp.329-336.”*

*Also, I think it should be highlighted that the same data has been used for comparison to CNNs (at the category-ROI level) in several previous studies.*

Thank you for pointing that out. We have added the above mentioned information. Please find the excerpts from the revised manuscript below.

**Excerpts from revised manuscript Page 14, Lines 412 to 415**

*“Using the same fMRI dataset as used in this study, a previous study^17^ showed that representation in scene-selective ROIs consists of both location and category information using scene-parsing DNNs.”*

*LANGUAGE/TYPOS*

*Lines 213-214: the ‘suggesting a function related to’ here sounds a bit naïve, as if no-one every thought about the function of these ROIs before; ‘consistent with’ may be more appropriate here.*

We respectfully disagree. By the above mentioned phrase we mean that our particular findings suggest a function related to a particular DNN task in an independent manner. While consistent in broad terms, the interpretation of our findings are independent of the previous research. In the discussion, we put our independent findings in context with the previous research. There we state that our findings are consistent with previous studies.

*Lines 284-287: I found the distinction between ‘quantitative’ and ‘qualitative’ not very helpful here: to me it seems all these analyses are quantitative? The distinction is that one shows unique variance, and the other does not, so it just seems like a ‘stricter’ criterion perhaps?*

We agree that the distinctions between the two analyses were not clear and the distinction using quantitative vs qualitative was not helpful. We have clarified the text following the reviewer’s suggestion. Please find the excerpt from revised manuscript below:

**Excerpts from revised manuscript Page 10-11, Lines 310 to 319**

*“First, in the functional map here, we highlighted the searchlight where one DNN group explained significantly higher variance uniquely than other 2 DNN groups. In the functional map of Figure 1d, we highlighted the DNN that explained the highest variance of a searchlight without performing any statistical analysis whether the selected DNN was significantly better than the second best DNN or not due to higher number of comparisons. Second, here we compared functions using groups of DNNs (3 functions: 2D, 3D and semantic), whereas in the previous analysis we compared functions using single DNNs (18 functions). The comparison using groups of DNNs allows us to put our findings in context with previous neuroimaging findings that are typically reported at this level.”*

*Lines 288-291: The use of ‘group level’ here is a bit confusing, I think it refers to ‘groups of DNNs’ but since it is followed by a statement about ‘typical reporting in neuroimaging analysis’ this might be interpreted as reflecting subject-group level.*

Thanks for pointing this out. We have clarified it in the revised manuscript.

**Excerpts from revised manuscript Page 11, Lines 315 to 319**

*“ Second, here we compared functions using groups of DNNs (3 functions: 2D, 3D and semantic), whereas in the previous analysis we compared functions using single DNNs (18 functions). The comparison using groups of DNNs allows us to put our findings in context with previous neuroimaging findings that are typically reported at this level.”*

*Lines 358: ‘revealed new functional insights for 15 ROIs’: again, what are these insights, and how new are they?*

We have updated the discussion section to list the new functional insights. Please find them above in response to your related comment about what new hypotheses are generated from our study.

*Lines 365: ‘both embody a theoretical hypothesis for explanation and predict brain responses well’ this sounds nice, but I don’t quite understand what is meant here.*

We thank the reviewer for providing us the opportunity to clarify. We have updated the sentence making it clearer. Please find relevant excerpts below.

**Excerpts from revised manuscript Page 16, Lines 474 to 476**

*“Our findings, thus, make advances towards finding models that generate new hypotheses about potential functions of brain regions as well as predicting brain responses well.”*

*Overall: in several places the word ‘the’ is missing before e.g., ‘model RDM’.*

Thanks for pointing this out. We have corrected it everywhere in the revised manuscript
